# Studying the Relationship between Robustness against Mutations in Metabolic Networks and Lifestyle of Organisms

**DOI:** 10.1155/2013/615697

**Published:** 2013-11-14

**Authors:** Sayed-Amir Marashi, Hawa Kouhestani, Majid Mahdavi

**Affiliations:** ^1^Department of Biotechnology, College of Science, University of Tehran, Tehran 1417614411, Iran; ^2^School of Computer Science, Institute for Research in Fundamental Sciences (IPM), P.O. Box 19395-5746, Tehran, Iran; ^3^Department of Biology, Faculty of Natural Science, University of Tabriz, Tabriz 5166616471, Iran

## Abstract

Robustness is the key feature of biological networks that enables living organisms to keep their homeostatic state and to survive against external and internal perturbations. Variations in environmental conditions or nutrients and intracellular changes such as genetic mutations have the potential to change stability and efficiency of an organism. Structural robustness helps biological systems to choose alternative routes of adaptation to varying conditions. In this study, in order to estimate the structural robustness in metabolic networks we presented a novel flux balance-based approach inspired by bond percolation theory. Fourteen *in silico* metabolic models were studied in this work in order to examine the possible relationship between the lifestyle of organisms and their metabolic robustness. The results of this study confirm that in organisms which are highly adapted to their environment robustness to mutations may decrease compared to other organisms.

## 1. Introduction

During the last years, many researchers have studied the robustness of metabolic networks against random mutations (for a recent review, please see [1]). The purpose of these studies is to investigate the mechanisms of protection of metabolic networks against mutations and to measure the tolerance of mentioned networks against “faults” (and maybe targeted attacks).

Robustness is defined as the “insensitivity” of a system to parametric variations [2]. Variation in parameters occurs by changes in the environmental conditions or by internal alterations [3, 4]. Structural robustness is an intrinsic property of most biological networks. Measuring the robustness of metabolic networks against mutations and gene/reaction deletion is an important question in systems biology [1]. Robustness in a metabolic network is a result of redundancy in metabolic pathways. The reason is that deficiencies in the network cannot be tolerated unless new alternative pathway(s) are evolved [5]. This is typically done by gene duplications [6] or by horizontal transfer of metabolic genes [7]. If alternative metabolic pathways are not present in a metabolic network, for example, due to reductive evolution [8, 9], then the metabolic network becomes extremely fragile [10]. It has been shown that metabolic networks are exceptionally robust when compared to appropriate null models [11].

In the present work, we introduce a novel approach to the analysis of metabolic network robustness. We study the resistance of metabolic networks to deletion of reactions by removing reactions until no flux can pass through the network. We show that eukaryotes and free-living prokaryotes show much higher mutational robustness compared to organisms which are highly adapted to their habitats.

## 2. Materials and Methods

### 2.1. Genome-Scale Metabolic Network Models

The genome-scale metabolic network models of 14 species are used in this study, including 3 eukaryotes (group 1), 6 “free-living” prokaryotes (group 2), and 5 prokaryotes with highly specific growth conditions (group 3) [12–20, 22, 24, 26, 28, 30]. Detailed information about the models is presented in Table 1.

### 2.2. Constraint-Based Analysis of Metabolic Networks

We used constraint-based analysis of metabolic networks in our study (for a brief review, please see Chapter  1 in [31]). In this modeling strategy, it is often assumed that steady-state conditions hold. Therefore, for a certain distribution of reaction fluxes, say **v**, the metabolic concentrations do not change during time. In a metabolic network with *m* metabolites and *n* reactions, this assumption is equivalent to the following equation:

(1) $$\begin{array}{c} S\cdot v=0, \end{array}$$

where **S** is an *m* × *n* matrix representing stoichiometric coefficients of metabolites in the reactions, **v** is the vector of the *n* steady-state fluxes, and 0 is an *m*-dimensional zero vector. Blocked reactions [32] are those reactions which cannot carry any nonzero flux. In other words, for a blocked reaction *i*, we have *v*_*i*_ = 0 subject to stoichiometric constraints (**S** · **v** = 0) and reversibility constraints (*v*_*j*_ ≥ 0 for all irreversible reaction like *j*). Finding blocked reactions is typically the first step of flux coupling analysis [32, 33]. In our study, we utilized F2C2 tool [34] for this purpose (see below).

### 2.3. Measuring Robustness

Our algorithm is inspired by the concept of percolation. For more information, the interested reader may refer to [35, 36]. Here, we briefly present the main idea of the percolation theory by an example.

**Figure 1:**
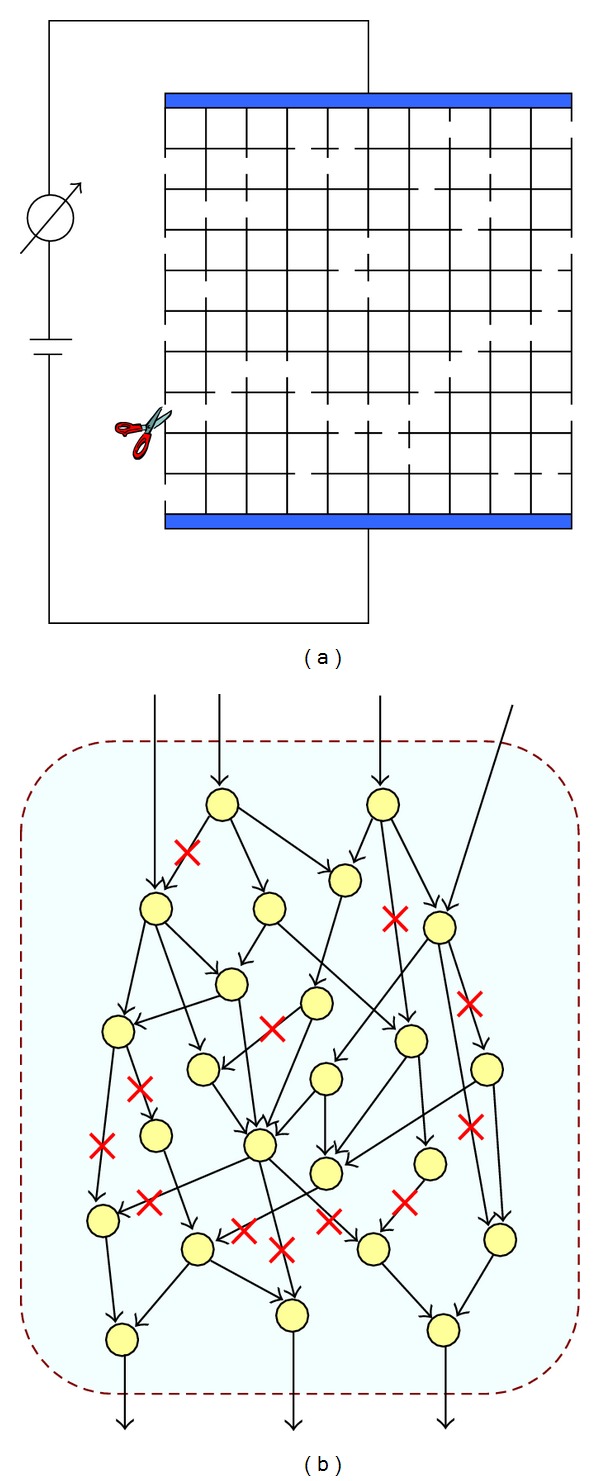
(a) Schematic representation of the two-dimensional bond percolation experiment. Conductivity of a steel-wire mesh is studied during the procedure of cutting wires (bonds) in the mesh. The goal is to find an average number of bonds which should be removed until the mesh conductivity vanishes to zero. (b) Schematic representation of our procedure. In each iteration of the modeling, a reaction is knocked out until no reaction can carry a nonzero flux in steady-state conditions.

Figure 1(a) shows a schematic representation of the Watson-Leath experiment [37]. Suppose that we have a two-dimensional steel-wire mesh (lattice). Two copper electrodes with negligible resistance are soldered to the two opposite sites of this square lattice. The resistance of the steel mesh is measured externally.

In each iteration of the experiment, a steel wire (a “bond”) is cut (Watson and Leath actually studied “site” percolation; i.e., in each iteration they cut the four wires coming to a junction). The electric conductance of the lattice gradually decreases by cutting the wires. The idea is to cut steel wires randomly until no electrical current can pass through the mesh.

Let *P* be the ratio of unblocked bonds to the total number of bonds. On average, when bonds are cut, at a critical value, say *P*_*C*_, conductivity of the lattice vanishes to zero [35]. Therefore, *P*_*C*_ is a random variable which can be estimated by repeating the experiment several times.

The method used in present study is based on solving a sequence of linear programming (LP) problems. In our algorithm, we used F2C2 [34] to study reactions deletions and their consequences on the activity of metabolic fluxes. The algorithm starts by correcting reversibility of reactions in a metabolic network and deleting all dead-end reactions. Then, in each iteration, one column of the stoichiometric matrix of the metabolic network (or equivalently, a reaction in metabolic network) is randomly deleted (Figure 1(b)). The procedure continues until all reactions become blocked based on the F2C2 program. Finally, the critical ratio is computed as follows:

(2) PC=Number  of  deleted  reactionsNumber  of  unblocked  reactions  in  the  original  network.

The experiment is repeated 100 times for each network, and average *P*_*C*_ values were computed for each of the metabolic network models.

We also compared our results with a classical measure of metabolic network robustness [38] based on flux balance analysis (FBA) [39]. This approach is based on *in silico* deletion of reactions. In each iteration, a reaction is deleted from the network and the sensitivity of the growth rate to the reaction deletion is modeled. We used the core reductive algorithm [8, 9, 40] for this purpose. In each iteration, we find a (randomly selected) minimal reaction set which can be used to produce biomass from a minimal growth medium in steady-state conditions. In a highly robust network, a considerable number of reactions can be deleted without influencing growth, while in a sensitive network deletion of a few reactions can result in no biomass production. Therefore, the average ratio of “unnecessary” reactions to the total number of reactions can be used as a measure of network robustness. For each metabolic network, the experiment was repeated 1000 times to have a good estimation of this ratio.

### 2.4. Statistical Analysis

The *R* package (http://www.r-project.org/) was used for statistical analyses. In order to compare the *P*_*C*_ distributions in two organisms, one-sided two-sample *t*-test was used. To investigate the correlation between *P*_*C*_ values and the number of reactions in the models, Pearson's product-moment correlation test was applied.

## 3. Results and Discussion

*P*_*C*_ was defined as the critical ratio of the fluxes to be removed such that a metabolic network becomes entirely blocked (Figure 1(b)). The higher the average *P*_*C*_ is, the higher the number of nonessential reactions is. Thus, we chose *P*_*C*_ to estimate the robustness of the metabolic networks.

**Figure 2:**
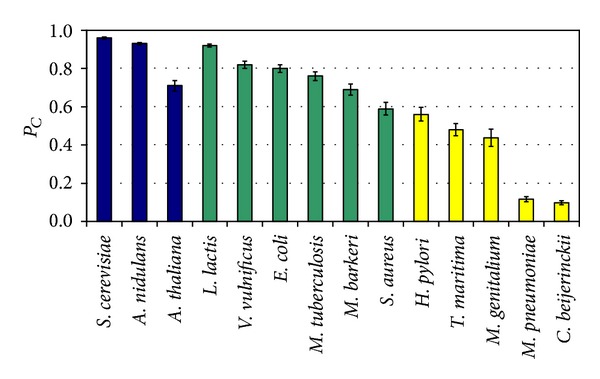
Average *P*_*C*_ for the fourteen metabolic network models. The histograms for group 1 (eukaryotes), group 2 (“free-living” prokaryotes), and group 3 (highly-adapted prokaryotes) are shown in dark blue, green, and yellow, respectively. The error bars in this plot are the 95% confidence intervals based on one-sample *t*-test.

Each set of deleted reactions is a cut set for the network [41] (but presumably not a minimal cut set). Therefore, the average *P*_*C*_ is an estimate for the average cut set size. For each of the fourteen metabolic networks in our dataset, we computed average *P*_*C*_ by repeating the reaction deletion procedure 100 times. The results of this analysis are summarized in Figure 2. From this figure, one can observe that there is comparable range of *P*_*C*_ values for metabolic networks in group 1 and group 2. However, for group 3, we face a range of *P*_*C*_ values which does not overlap with the range of *P*_*C*_ values for groups 1 and 2. This observation implies that the network robustness of group 3 is much less than that of groups 1 and 2.

**Figure 3:**
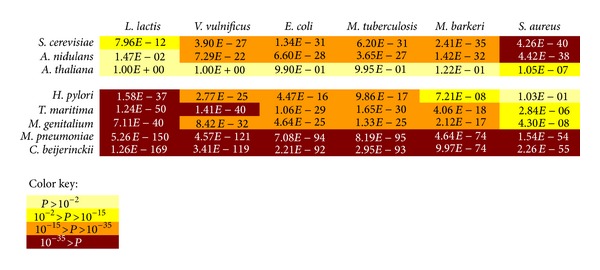
The upper box shows the *P* values of *t*-test for *P*_*C*_ (group 1) > *P*_*C*_ (group 2), while the lower box shows the *P* values of *t*-test for *P*_*C*_ (group 2) > *P*_*C*_ (group 3).

To investigate the significance of differences between *P*_*C*_ values of different groups, one-sided two-sample* t*-test was used. We tested whether the *P*_*C*_ values of eukaryotes (group 1) are significantly greater than *P*_*C*_ values of free-living prokaryotes (group 2) and whether the *P*_*C*_ values of free-living prokaryotes (group 2) are significantly greater than *P*_*C*_ values of prokaryotes with highly specific growth conditions. The results are summarized in Figure 3. Obviously, the differences between group 2 and group 3 are much more significant than the differences between group 1 and group 2. This observation confirms that prokaryotes with highly specific growth conditions are significantly less robust than the free-living prokaryotes.

We also tested whether the number of unblocked reactions (and not the network structure) determines the network robustness. We found that although the correlation between the number of reactions and *P*_*C*_ is positive (*R* ^2^ = 0.26), it is not statistically significant (*P* value > 0.05 in Pearson's product-moment correlation test). Therefore, there is only a weak, if any, relationship between the number of reactions and network robustness. This finding emphasizes the importance of the network “structure” and “wiring” (as an intrinsic property in each metabolic network) in shaping the network mutational robustness.

In this study, group 1 includes eukaryotic species (*S. cerevisiae*, *A. nidulans*, and *A. thaliana*). It is well known that *S. cerevisiae* and *A. nidulans* can adapt to a wide range of growth conditions. Moreover, *A. thaliana* is a multicellular organism with different tissues. For these reasons, in group 1 we expect a large number of alternative metabolic pathways, which in turn results in great robustness values. It should be noted that, in the metabolic model of *A. thaliana*, only 672 unblocked reactions are included. However, due to the large number of metabolic enzymes involved in the plants, this number is greatly underestimated. One expects that addition of the missing metabolic pathways to this model will greatly enhance the robustness of this network.

In group 2, we have six prokaryotes which are able to grow in different habitats. *L. lactis* is well known for its application to the lactic industry, while it is reported that this species is also isolated from vegetables [42] and intestinal tract of the Amur catfish [43]. On the other hand, *V. vulnificus*, which is a cause of deadly food poisoning and wound infections, is also present in brackish ponds [44]. Another species from this group, that is, *E. coli*, can easily grow in human intestine, in water, and in soil [45]. While *M. tuberculosis* is a human pathogen, it is proven that this bacterium has an exceptional ability to survive environmental stresses [46]. Moreover, it can grow in different growth conditions, both *in vivo* and *in vitro* [47]. *S. aureus* is typically known as a human pathogen. However, there is a growing body of evidence that this microorganism is also able to grow in a variety of different conditions, including foods [48] and soil [49]. *M. barkeri* is the only archaeon in this group, with the ability to grow in many different growth conditions ranging from rumen [50] to freshwater lagoons [51].

Group 3 includes bacterial species with highly specific growth conditions. *H. pylori*, a human pathogen, grows in highly acidic environment of the stomach [21], *T. maritima* only grows in extremely thermophilic conditions [23], and *C. beijerinckii* grows only in strictly anaerobic conditions [29]. *M. genitalium *and *M. pneumoniae*, on the other hand, have reduced metabolic networks which helps them to grow faster as intracellular pathogens [25, 27]. The extreme level of adaptation in species of group 3 has resulted in the greater degree of “nonrobustness” in the metabolic networks of these organisms.

In this work, we presented a novel way of measuring metabolic network robustness in the constraint-based modeling framework. Another class of robustness measures in this framework is based on FBA [38]. We believe that our robustness measure is more suitable for comparing a variety of living organisms. The reason is that FBA is always based on a predefined objective function (e.g., biomass production flux) and a predefined growth medium (i.e., those uptake reactions which can take nonzero flux values), which can vary across species. Despite this fact, all measures of robustness must be correlated. For example, a highly robust metabolic network with many alternative pathways is identified as a robust network by any measure of robustness.

In order to find the relationship between our novel robustness measure and the FBA-based measure of robustness, we used our recent implementation of the core-reductive algorithm [40] to obtain minimal metabolic subnetworks which are able to produce biomass. The number of reactions which can be deleted without decreasing biomass production is an FBA-based measure of network robustness. We found out that the results are qualitatively comparable, with a relatively high correlation between the two measures (Pearson's correlation *R* = 0.80). The results confirm that our novel robustness measure is comparable with the classical measures of robustness, but with the advantage that no additional assumption is required for its computation.

## Figures and Tables

**Table 1:** List of species used in the present work.

	Species name	Specific growth conditions	Metabolic network ID	Number of reactions	Network reference
Eukaryotes	*Saccharomyces cerevisiae *	N/A	iIN800	1292	[12]
	*Aspergillus nidulans *	N/A	iHD666	711	[13]
	*Arabidopsis thaliana *	N/A	AraGEM	672	[14]

“Free-living” prokaryotes	*Lactococcus lactis *	N/A	—	671	[15]
	*Vibrio vulnificus *	N/A	VvuMBEL943	642	[16]
	*Escherichia coli *	N/A	iAF1260	2167	[17]
	*Mycobacterium tuberculosis *	Human pathogen	iNJ661m	800	[18]
	*Methanosarcina barkeri *	Diverse anaerobic conditions	iAF692	538	[19]
	*Staphylococcus aureus *	Human pathogen	iSB619	583	[20]

Prokaryotes with highly specific growth conditions	*Helicobacter pylori *	Extremely acidic conditions [21]	iIT341	501	[22]
	*Thermotoga maritima *	Extremely thermophilic conditions [23]	—	547	[24]
	*Mycoplasma genitalium *	Intracellular conditions [25]	iPS189	46	[26]
	*Mycoplasma pneumoniae *	Intracellular conditions [27]	iJW145	300	[28]
	*Clostridium beijerinckii *	Strictly anaerobic conditions [29]	iCB925	501	[30]
